# The Development of Relational Reasoning in South Korean Elementary and Middle-School Students: A Cross-Sectional Investigation

**DOI:** 10.3389/fpsyg.2021.630609

**Published:** 2021-03-04

**Authors:** Soo Eun Chae, Patricia A. Alexander

**Affiliations:** ^1^Department of Teacher Education, Gangneung-Wonju National University, Gangneung, South Korea; ^2^Department of Human Development and Quantitative Methodology, University of Maryland, College Park, MD, United States

**Keywords:** relational reasoning, analogy, anomaly, antinomy, antithesis

## Abstract

Relational reasoning is a higher-order executive function that involves the ability to perceive meaningful patterns within a body of seemingly unrelated information. In this study, the ability of 749 fourth (*M*_*age*_ = 10), sixth (*M*_*age*_ = 12), eighth (*M*_*age*_ = 14), and tenth graders (*M*_*age*_ = 16) to identify meaningful relational patterns was investigated. This general cognitive ability was assessed by means of the Test of Relational Reasoning-Junior (TORRjr), a 32-item measure organized into four 8-item scales that assess analogical, anomalous, antinomous, and antithetical reasoning. Students’ performance on the TORRjr was analyzed using confirmatory factor analysis, measurement invariance test, and non-parametric median-based analyses. The confirmatory factor analysis supported that the higher-order factor model was the best fit for the TORRjr data for the Korean students. The measurement was determined to be invariant by gender but variant across grade levels. The non-parametric analysis resulted in an asymptotic (a constant increasing up to grade 6 and then a level off witnessed from grades 8 to 10) development pattern in overall relational reasoning across the grades. In comparison to analogy and anomaly, antinomy and antithesis scores were more fully developed by grade 8 and that level of performance was maintained at grade 10. The TORRjr appeared to be a viable measure for the Korean samples up to approximately 15 years of age. The significance of these findings for research and instructional practice are discussed.

## Introduction

*Relational reasoning* is a higher-order cognitive ability to perceive meaningful patterns within a body of seemingly unrelated information (Alexander and The Disciplined Reading and Learning Research Laboratory, 2012; Diamond, 2013; Dumas et al., 2013). So defined, relational reasoning has been shown to play a crucial role in learning and performance for individuals of varying ages and across different contexts (Dumas et al., 2014; Jablansky et al., 2016). For example, studies have shown that relational reasoning is evident in activities that entail both formal and informal learning (Galotti, 1989; Barwise, 1993) and manifests in such disciplines as medicine (Greenwood and King, 1995), engineering (Murphy et al., 2017), science and mathematics (Alexander, 2017; Resnick et al., 2017), reading (Kendeou et al., 2017), and writing (Egyed, 2010). There is a growing interest in relational reasoning, fueled in part by contemporary research in cognitive neuroscience (Baggetta and Alexander, 2016; Wertheim and Ragni, 2018; Gray and Holyoak, 2020) and in educational and cognitive psychology (e.g., Grossnickle et al., 2016; Jablansky et al., 2019). However, recognition of its importance goes back decades to work by James (1890), Spearman (1927), Cattell (1949), and others. For example, in his *Principles of Psychology*, William James (1890) described the ability to discriminate differences and similarities as essential to human thinking and learning. Pattern recognition was also central to Cattell (1949) Culture Free Intelligence Test. Given the weight of the contemporary and historical evidence documenting the significance of relational reasoning to cognitive performance, it is justified to investigate the initial manifestation of relational reasoning and how it develops over time. Indeed, questions about relational reasoning have garnered attention in the research of both children (Goswami, 1989, 2013; Richland et al., 2006; Jablansky et al., 2016) and adults (Alexander et al., 1989; Holyoak, 2012). The resulting body of research has afforded insights into when relational reasoning emerges and how it changes over the lifespan (Diamond, 2006, 2013). Still, significant gaps in theory and research on the course of relational reasoning development remain. With regard to onset, for instance, there is evidence that even children as young as four can manifest relational reasoning when the conditions and contexts are facilitative (White and Alexander, 1986; Chiu and Alexander, 2014). Those facilitative conditions include familiarity with the content or task, scaffolding or feedback from a teacher or more knowledgeable other, and a task environment that is interesting and motivating (Marzolf et al., 1999). However, in the aforementioned studies, only one form of relational reasoning was investigated, analogical reasoning, which pertains to the discernment of associations based on similarities.

### Test of Relational Reasoning-Junior

In recent years, efforts have been made into the development of fluid measures of relational reasoning that go beyond analogical reasoning. For one, Alexander and The Disciplined Reading and Learning Research Laboratory (2012) set out to create a fluid measure that assessed analogical, anomalous, antinomous, and antithetical reasoning in figural form. According to this investigation, analogical reasoning signifies recognition of similarities among objects or information. Anomalous reasoning is an ability to detect an exceptional case in certain groups of objects. With antinomous reasoning ability, one can identify paradoxical situation necessitating acceptance of two or more ideas that appear contradictory. Antithetical reasoning means an ability to detect exact opposite of a certain procedure or a concept. As a result of the extended conceptualization of relational reasoning, the DRLRL developed the Test of Relational Reasoning (TORR), a 32-item measure consisting of four 8-item scales, each targeting one form of relational reasoning. Although normed and standardized on adolescent and adult samples in the United States, the TORR has been administered globally, including in Israel (Aharon and Eilam, 2019) and Russia (Federiakin and Aleksandrova, 2017), with similar outcomes in terms of factor structure, reliabilities, and item functioning. This suggests that the examination of relational reasoning, at least by means of fluid measures like the TORRjr, may be less susceptible to cultural differences. Of course, more research on the effects of social and cultural factors on relational reasoning is required.

While the TORR fills the need for an alternative measure that captures the multiple manifestations of relational reasoning of adolescents and adults, it does not address the assessment needs for a younger population. For that reason, Alexander and the DRLRL created Test of Relational Reasoning-Junior or TORRjr, a parallel but easier version of the TORR that was specifically for elementary and middle-school students (approximately grades 3 to 7). Earlier versions of the TORRjr, have been administered to elementary- and middle-school students in the United States, New Zealand, and Israel (Alexander and Jablansky, 2017; Jablansky et al., 2017). The final version of the TORRjr was recently validated and standardized using data from 790 Chinese students in grades 3 to 7 (Zhao et al., 2020).

### Changes in Relational Reasoning Over the Lifespan

Based on more limited research (Jablansky et al., 2016, 2019), the developmental trajectory for the multiple forms of relational reasoning appears to vary over time. For one, they conducted a longitudinal study based on the frequency of students’ relational reasoning utterances when explaining the design and “fit for purpose” of both familiar and novel technological devices (i.e., juice box and vegetable cutter). Participants were a nationally representative cross-sectional sample of 61 New Zealand primary and secondary students, divided into three grade groups: early (pre-kindergarten through second), middle (fourth through eighth), and late (tenth through eleventh). Results indicated that children as young as 5 years old were capable of using all 4 forms of relational reasoning in discourse. However, Jablansky et al. (2016, 2019) found that analogical reasoning and anomalous reasoning utterances (i.e., the recognition of aberrance) were dominant in the problem solving of the younger students (ages 5 to 10), than antinomous reasoning (i.e., determinations of exclusivity) and antithetical reasoning (i.e., discernment of opposition). However, antinomous and antithetical reasoning were more likely to be present in the utterances of 15- to 17-year-olds’ problem solving.

In contrast, in their developmental study involving 148 females, ages 7 to 30, Dumontheil et al. (2010) reported a dip in relational reasoning development in an otherwise linear growth trajectory during mid-adolescence. Yet, Dumontheil et al. (2010) investigated only analogical reasoning and included only females. In contrast, Jablansky et al. (2016) involved both males and females and focused on the four forms of relational reasoning (i.e., analogy, antinomy, antinomy, and antithesis). Indeed, what might account for the differential patterns for the distinct forms of relational reasoning over time remains an open question. Nonetheless, we would hypothesize that these varied patterns for the forms of relational reasoning by age groups could reflect greater familiarity for analogy and anomaly than antinomy and antithesis, as well as the process required to discern and then map the pattern associated with true categorical distinctions (antinomies) vs. oppositional differences (antithetical).

### Research Gaps

Despite the existent literature on the onset and lifespan changes of relational reasoning to date, several gaps in the extant knowledge remain to be addressed. For one, relational reasoning is broadly conceptualized to include relations based on similarities and dissimilarities (James, 1890; Cattell, 1949; Alexander et al., 2016). Nonetheless, it has been rather narrowly operationalized (Dumas et al., 2013). As noted, most relational reasoning studies have measured or observed only analogical reasoning (e.g., McGivern et al., 2002; Richland et al., 2006). Thus, many of the conclusions that researchers have reached about relational reasoning are most often solely about analogical reasoning and overlook other forms of relational reasoning that deal with dissimilarities, which may be more cognitively demanding.

Moreover, one research gap in understanding persis around relational reasoning is the paucity of sound measures or experimental tasks that allow for its full assessment, particularly in culturally diverse populations. For instance, in cognitive science and neuroscience (Thibaut et al., 2010; Dumas et al., 2014), the Raven’s Progressive Matrices (Raven, 2003), which is a fluid measure of cognitive ability, remains the gold standard for relational reasoning assessment. By definition, a fluid assessment is a novel and typically nonlinguistic measure for which all necessary information for solution is contained in the problem; thus making it more culturally fair than crystallized intelligence tests containing culturally specific information (Kidd, 1962). The figural test forms in Raven’s Matrices do not necessitate knowledge acquired through formal schooling in any particular country or culture (Sternberg and Grigorenko, 2006).

Despite its frequent use, there are shortcomings to the Raven’s as a measure of relational reasoning. Thus, while novel and more culturally fair, the Raven’s is constructed entirely of matrix analogy problems. Other forms documented in the literature are unaddressed. Also, the brain activities of individuals solving select Raven’s items are typically registered by neuroimaging techniques (e.g., Dumontheil et al., 2010; Wertheim and Ragni, 2018; Gray and Holyoak, 2020). The expertise, funds, and facilities that these neuroimaging techniques require render them impractical for more pedestrian and widespread use in relational reasoning research. In addition, the appropriateness of these neuroimaging techniques for assessing the thinking and reasoning of young children is debatable due to their potential harm to mental health (Burke, 1958; Mills and Tissot, 1995). Capturing brain activities using neuroimaging techniques can be another way to investigate relational reasoning although this methodology is known economically inefficient and clinically harmful.

Others invested in researching the onset and changes in relational reasoning have employed alternative measurement tools. Along with more traditional verbal and figural analogy tests (e.g., Miller Analogy Test; Murray, 1979), these alternatives have included reasoning problems cast as scenarios or stories or have positioned relational reasoning assessment in a game-playing context (Gick and Holyoak, 1980; Alexander et al., 1986; Chen, 1996; Goswami, 2013). When only analogical reasoning is assessed and when the measures are linguistic in nature, then the ability to uncover relational reasoning within non-Western or non-English-speaking populations is constrained. It is constrained both by the exclusion of other relational reasoning forms (e.g., antithetical reasoning) and by demands on verbal and socio-cultural knowledge that may not be accessible to those populations.

### The Present Study

Given these research gaps, further investigations into young children’s relational reasoning in non-western countries are warranted to expand current understanding. Thus, the present study was undertaken to build on Zhao et al. (2020) work in several ways. For one, we set out to administer the TORRjr to a sample of Korean children in grades 4, 6, 8, and 10; a population that has not been previously tested. Our goals were to explore the changes in relational reasoning overall and by form for this age range. Further, the grade levels included in this study extend beyond those for which the TORRjr was initially devised, allowing us to test the upper limits of the TORRjr.

The specific research questions guiding this investigation and the hypothesis were as follows:

1. What do findings for Korean students in grades 4, 6, 8, and 10 reveal about the psychometric properties and factor structure of the TORRjr?

Based on the performance of the TORR (Dumas et al., 2014; Dumas and Alexander, 2018) and the recent study of the TORRjr with Chinese students (Zhao et al., 2020), we hypothesized that data from the TORRjr would be found to be psychometrically sound for students in grades 4 to 6. It remains unclear how the TORRjr will function for students in grades 8 and 10, given that these students are older than those for whom the TORRjr was originally developed.

2. How do grade level and gender affect students’ relational reasoning performance, as measured by the TORRjr?

We expected that grade level is a determinant of students’ relational reasoning performance. In prior investigations involving populations of primary, elementary, and middle-school students and using various research methodologies and data sources, evidence emerged that certain forms of relational reasoning are earlier developing, specifically analogical and anomalous reasoning (Jablansky et al., 2016, 2019; Zhao et al., 2020).

In contrast to grade level, the gender effects remain an open question with regard to the TORRjr. On the one hand, the TORR has been found to be invariant with regard to age, gender, and ethnicity for college-age students (Dumas and Alexander, 2018). On the other hand, Zhao et al. (2020) found a difference in TORRjr performance, with girls scoring higher than boys at grade 7. Thus, the findings from the current investigation may serve to corroborate or disconfirm the gender effect reported by Zhao et al. for Chinese children.

3. To what extent is TORRjr a psychometrically appropriate measure of relational reasoning for older students (grades 8–10) compared to younger students (grades 4–6)?

With the inclusion of students from grades 8 and 10 in the current investigation, there was the opportunity to determine whether the TORRjr, which was originally developed for students in grades 3 to 7, would result in scores that were still reliable for these older students. Without data from prior studies upon which to rely, however, we cannot forward a prediction on the suitability of the TORRjr, especially for students in grade 8 who are at the cusp of the recommended grade range.

4. What trends can be discerned in the overall TORRjr performance of the students in grades 4, 6, 8, and 10, and do these trends vary for the four individual scales?

A different path of the developmental trajectory was expected varying forms of TORRjr. The previous studies using the same measure (Zhao et al., 2020), the examination of adolescents’ relational reasoning utterances (Jablansky et al., 2016), or investigation using brain imaging techniques (e.g., Dumontheil et al., 2010) have commonly shown that reasoning performances show steep development by approximate age of 15 and later leveled off. However, studies incorporating all four forms of relational reasoning (e.g., Jablansky et al., 2016, 2019; Zhao et al., 2020) indicated early development of analogical and anomalous reasoning compared to that of antinomous and antithetical reasoning.

## Materials and Methods

### The Educational Context

All the students who participated in this study were from elementary, middle, and high schools in South Korea. Formal education in South Korea is referred to as the “6-3-3 schooling system,” signifying 6 years of elementary school, 3 years of middle school, and 3 years of high school, with mandatory education beginning at age 7. When students enter middle school in seventh grade, they are approximately 13 years of age. In addition, the school year in South Korea begins in March, meaning that the participants from this study are older than United States students at the same grade level. The elementary schools and middle schools in South Korea follow the principle of equalized allocation. That means that there is heterogeneity concerning academic ability in elementary and middle-school classrooms (Korean Educational Development Institute, 2018).

In contrast, there are four types of high schools in South Korea that differ in their educational aims: public high schools, special-purpose high schools, self-governing high schools, and specialized high schools (i.e., “Meister” schools). The public high schools are academically heterogeneous, as are the elementary and middle schools. The educational purpose regards to cultivate the qualities of the democratic citizen who pioneer the career and to communicate with the world based on the achievement of middle school education (Korean Institute for Curriculum and Evaluation, 2015). Special-purpose, self-governing, and Meister high schools target students with special needs, such as gifted students or students who want to go to prestigious colleges and provide vocational training (Korean Educational Development Institute, 2018).

### Participants

The participants in this study were 749 fourth (*M*_*age*_ = 10), sixth (*M*_*age*_ = 12), eighth (*M*_*age*_ = 14), and tenth graders (*M*_*age*_ = 16) attending three schools (one school per school level) in Gangwon-do Province, South Korea. The area students were located in was a medium-sized city, mostly consisting of middle-class families. According to school records, all the participants were typical adolescents from the middle-class families. All the students attending the schools on the day of data collection were involved in the data collection. The gender distribution and ages of participants by grade level are presented in Table 1. Students in these grades were of particular interest to this study because this age range would afford a more comprehensive view of relational reasoning development. All participants, whether elementary, middle, or secondary students, pursued the public academic curriculum provided by the South Korean central government. All students who participated were included in the analysis, with the exception of five students who failed to respond to eight or more consecutive items on the TORRjr.

**TABLE 1:** Mean and SD by grade level and gender.

	Grade Level				
	
Variables	4	6	8	10	4 to 10
Boy	109 (55.33%)^1^	105 (52.5%)	81 (39.32%)	63 (43.15%)	358 (50.14%)^2^
Girl	87 (44.16%)	95 (47.5%)	117 (56.8%)	57 (39.04%)	356 (49.86%)
Total	196	200	206	146	749
*Mean* age	10	12	14	16	

*^1^The percentage is based on the subtotal number of the participants for each grade level. ^2^The percentage is based on the total number (*n* = 749) of the participants.*

### Measure

As discussed, the measure used to examine the changes in relational reasoning in this study was the TORRjr (Alexander and The Disciplined Reading and Learning Research Laboratory, 2019). While the original measure was developed in English, it has subsequently been translated into Hebrew, Arabic, and Chinese. For this investigation, the TORRjr had to be translated into Korean. Following a procedure used in prior studies, the English version was first translated into Korean by the first author and then back-translated by an English language professor experienced in translation but blind to the purpose of the study. The back-translated version was then compared to the original wording of the TORRjr, and discrepancies between the two versions were corrected.

**FIGURE 1:**
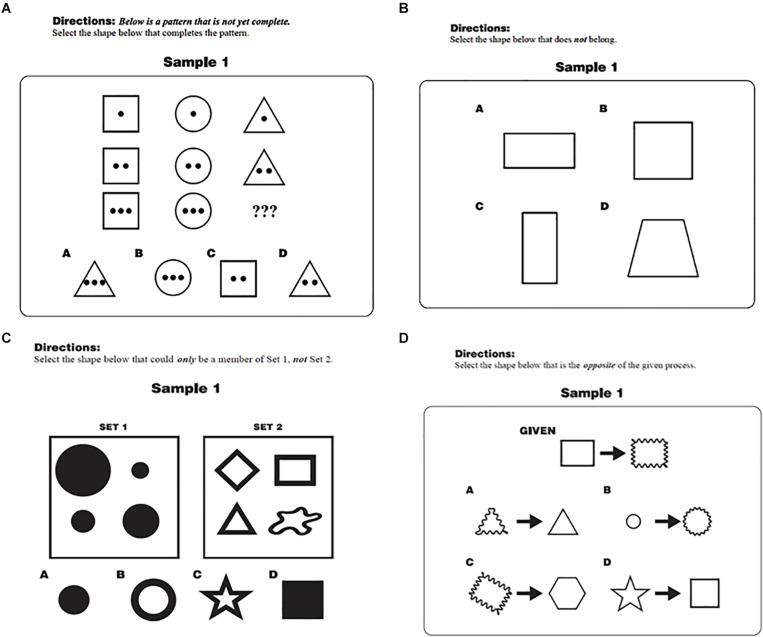
Sample items from the TORRjr for the **(A)** Analogy, **(B)** Anomaly, **(C)** Antinomy, and **(D)** Antithesis.

The Korean version of the TORRjr was presented in booklet form, preceded by a demographics sheet requesting students’ date of birth, gender, and grade level. This delivery format was deemed acceptable since no statistical differences have been found for paper vs. online versions (Jablansky et al., 2017). The four scales were presented in a fixed order (i.e., analogy, antinomy, antinomy, and antithesis), and each scale began with two sample items. No order effects were reported in previous studies based on random-ordered versions (Dumas and Alexander, 2016; Zhao et al., 2020). These sample items, which were not scored, were included to help students understand the directions and to reorient them as they moved to different scales. One sample item from each scale is displayed in Figure 1. For the analogy scale, for example (Figure 1A), students were directed to find the shape from the six options that completed the pattern shown. The second scale, antinomy, asked the students to identify the shape that did not fit the pattern (Figure 1B). The prompt for the antinomy scale (Figure 1C), which included two problem sets, directed students to find the shape from the six options that could belong to set A but not to set B. Finally, for the antithesis scale (Figure 1D), students were told to select the option that was the opposite of the process shown in the given problem.

### Procedure

The TORRjr was administered to students in their classrooms under the supervision of their classroom teacher. The testing took place in November and December 2018. The students were told that they had all the time they required to complete the test. However, all the students finished the test within a span of 40–45 min. Before the test, students’ and their parents’ written consent forms were collected.

## Results and Discussion

### Descriptive Statistics

Table 2 shows the average relational reasoning scores as a function of grade level and gender. The average of the composite relational reasoning scores is indicative of an asymptotic developmental trend that transcends the age groups. Specifically, the mean relational reasoning score increased to the grade 8 and remained at approximately the same level between the grades 8 and 10. Further, except for grade 10, the students performed best on the analogy scale followed by antinomy, anomaly, and antithesis, respectively. While the tenth graders also performed best on analogies followed by antinomy, their antithetical reasoning was better than their anomalous reasoning. The data distribution was normal for grade 4 but was found to be non-normal for higher grade levels.

**TABLE 2:** Means and SD for TORRjr total and scale scores by grade level and gender.

Grade Level	Relational Reasoning Form				
	
Gender	Total *M(SD)*	Analogy *M(SD)*	Anomaly *M(SD)*	Antinomy *M(SD)*	Antithesis *M(SD)*
4	19.89 (5.68)	6.07 (1.59)	4.57 (1.76)	5.42 (2.28)	3.83 (2.72)
Boy (*n* = 109)	20.03 (5.74)	6.23 (1.46)	4.62 (1.70)	5.28 (2.36)	3.91 (2.66)
Girl (*n* = 87)	19.66 (5.63)	5.85 (1.71)	4.52 (1.85)	5.60 (2.18)	3.69 (2.80)
6	21.30 (6.09)	6.70 (1.38)	5.30 (1.99)	5.46 (2.36)	3.84 (2.69)
Boy (*n* = 105)	19.91 (5.90)	6.47 (1.49)	4.90 (2.05)	4.94 (2.51)	3.60 (2.67)
Girl (*n* = 95)	22.82 (5.95)	6.96 (1.19)	5.74 (1.83)	6.03 (2.06)	4.10 (2.70)
8	24.34 (5.46)	7.01 (1.17)	5.73 (1.69)	6.35 (1.92)	5.26 (2.76)
Boy (*n* = 81)	23.25 (5.75)	6.86 (1.36)	5.48 (1.68)	6.00 (2.03)	4.90 (2.69)
Girl (*n* = 117)	25.33 (4.95)	7.14 (1.03)	5.93 (1.68)	6.68 (1.74)	5.59 (2.75)
10	23.70 (5.93)	6.69 (1.34)	5.23 (1.75)	6.21 (2.08)	5.58 (2.70)
Boy (*n* 63)	23.97 (6.21)	6.70 (1.49)	5.49 (1.67)	6.16 (2.18)	5.62 (2.57)
Girl (*n* = 57)	22.61 (6.09)	6.49 (1.33)	4.81 (1.78)	6.04 (2.12)	5.28 (2.89)
4 to 10	22.23 (6.05)	6.61 (1.42)	5.21 (1.85)	5.84 (2.21)	4.56 (2.82)
Boy (*n* = 358)	21.41 (6.12)	6.53 (1.47)	5.05 (1.83)	5.50 (2.35)	4.34 (2.75)
Girl (*n* = 356)	22.84 (5.95)	6.67 (1.40)	5.34 (1.87)	6.14 (2.03)	4.68 (2.88)

Specifically, data skewness ranged from −1.74 to 0.15, depending on the grade and the form of relational reasoning, with the kurtosis ranging from −0.82 to 3.9 (see Table 3). The skewness and kurtosis were non-significant for grade 4 but became more serious toward higher grade levels. McDonald’s omega for the internal reliability was 0.85 for grades 4–8, but dropped to 0.87 when all grade levels were included (grades 4–10).

**TABLE 3:** Skewness (S) and Kurtosis (K) by relational reasoning (RR) form and grade level.

RR	Grade Level				
	
Form	Total *S/K*	4 *S/K*	6 *S/K*	8 *S/K*	10 *S/K*
Total	2.10/−1.34	0.91/−0.97	3.80/−1.59	3.90/−1.74	0.65/−1.05
Analogy	−0.54/−0.48	−0.67/−0.09	−0.41/−0.66	−0.16/−0.66	−0.43/−0.6
Anomaly	0.01/−1.02	−0.53/−0.73	−0.34/−0.86	0.95/−1.35	0.65/−1.28
Antinomy	−1.43/−0.25	−1.44/0.07	−1.37/0.15	−1.04/−0.67	−0.77/−0.83
Antithesis	−0.74/−0.44	−0.81/−0.08	−0.82/−0.23	0.46/−0.92	−0.43/−0.82

Next, we checked item difficulties for each grade level (Table 4). Item difficulty in this study was the percentage of correct responses for each item, indicating the overall suitability of that item for the designated grades. Item difficulties between 0.20 and 0.80 are generally considered within an acceptable range (Kehoe, 1995). In this study, the item difficulties for the total sample (grades 4 to 10) ranged from 0.36 to 0.92. Those ranges at the specific grade levels were as follows: 0.31 to 0.89, grade 4; 0.38 to 0.93, grade 6; 0.37 to 0.95, grade 8; and 0.37 to 0.93, grade 10. However, it should be noted that only one item (Analogy #4) was found to be very easy for respondents in grades 6 and 10 (i.e., >0.90). This item is exclusively proper for the youngest student groups due to its extreme easiness for upper-grade levels. However, other items seem to be within an acceptable range of easiness for the participants at most grade levels. Only three easy items in Analogy were found for grade 10. No items in anomaly, antithesis, and antinomy were indicated as being extremely easy to students at every grade. These item difficulties indicated that the TORRjr was a little easier for this sample of South Korean children than had been reported for the norming sample of Chinese children in grades 3 to 7 (Zhao et al., 2020). The analogy scale, followed by the anomaly scale, appeared to be the easiest for students at every grade level.

**TABLE 4:** Item difficulties by relational reasoning (RR) form and grade level.

RR Form Item number	Total *n* = 754	Grade 4 *n* = 197	Grade 6 *n* = 200	Grade 8 *n* = 209	Grade 10 *n* = 148
Analogy					
1	0.79	0.73	0.76	0.87	0.81
2	0.78	0.74	0.81	0.78	0.80
3	0.68	0.57	0.72	0.75	0.66
4	0.91	0.82	0.93	0.95	0.93
5	0.89	0.84	0.89	0.93	0.88
6	0.85	0.82	0.85	0.89	0.85
7	0.85	0.79	0.88	0.88	0.84
8	0.86	0.75	0.89	0.93	0.89
Anomaly					
1	0.41	0.42	0.48	0.37	0.37
2	0.49	0.31	0.67	0.52	0.45
3	0.83	0.78	0.81	0.89	0.85
4	0.61	0.51	0.59	0.72	0.63
5	0.87	0.89	0.84	0.89	0.86
6	0.75	0.65	0.71	0.85	0.81
7	0.60	0.46	0.62	0.71	0.62
8	0.62	0.54	0.60	0.71	0.64
Antinomy					
1	0.74	0.65	0.69	0.83	0.79
2	0.75	0.71	0.71	0.82	0.76
3	0.75	0.75	0.71	0.78	0.76
4	0.68	0.64	0.60	0.74	0.73
5	0.68	0.63	0.69	0.71	0.70
6	0.80	0.76	0.75	0.85	0.87
7	0.62	0.53	0.57	0.70	0.72
8	0.79	0.76	0.76	0.83	0.81
Antithesis					
1	0.73	0.71	0.70	0.73	0.78
2	0.59	0.46	0.51	0.67	0.75
3	0.55	0.41	0.45	0.67	0.68
4	0.62	0.54	0.51	0.69	0.75
5	0.48	0.42	0.41	0.51	0.61
6	0.48	0.38	0.38	0.58	0.59
7	0.55	0.47	0.45	0.62	0.68
8	0.57	0.45	0.45	0.70	0.71
Minimum	0.41	0.31	0.38	0.37	0.37
Maximum	0.91	0.89	0.93	0.95	0.93

### Research Question 1: Factor Structure of the TORRjr

**FIGURE 2:**
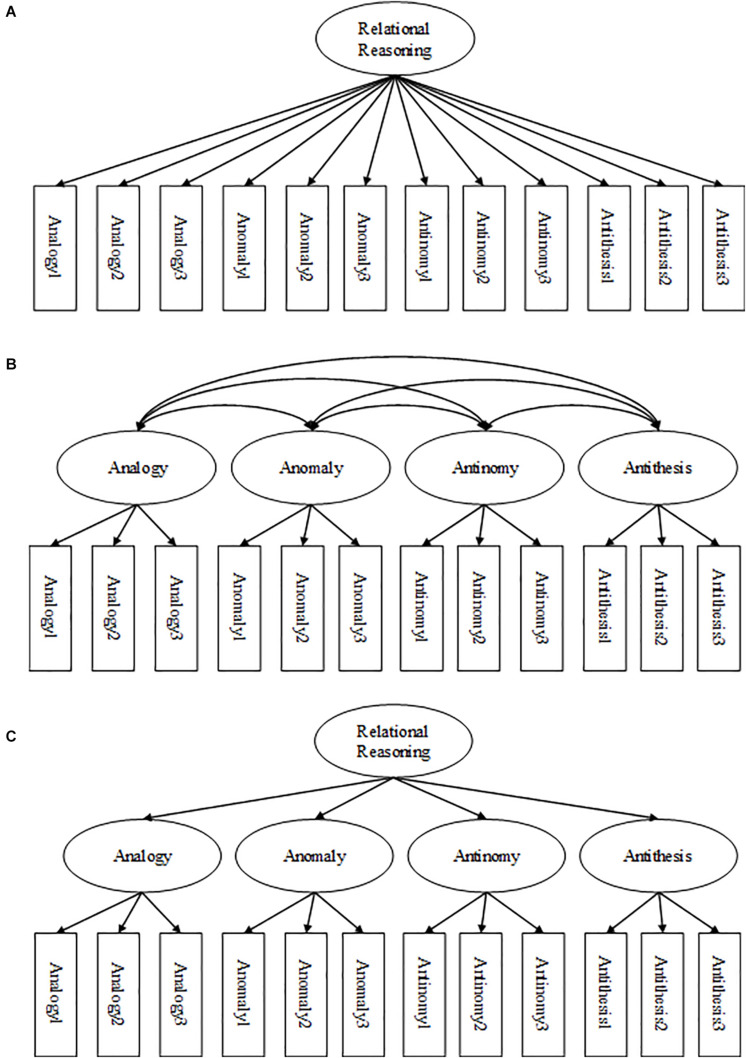
Factor structure models tested for the TORRjr: **(A)** one-factor model; **(B)** four-factor model; and **(C)** higher-order factor model. Only 3 of the 8 items entered in analyses are displayed.

To address psychometric properties and factor structure of the TORRjr in Korean students in grades 4, 6, 8, and 10, we tested the dimensionality of the data using latent factor analysis to determine the appropriate model fit (Holmes Finch and French, 2007). We tested three models that had been indicated by prior investigation (Figure 2). The first was a one-factor model, and the second was a four-factor correlated model. In the one-factor model, relational reasoning was assumed to load onto each item. In the four-factor model, relational reasoning was theorized to consist of four separate, but related, latent constructs corresponding to each of the four scales. The third was a higher-order model found to fit TORRjr data best in the norming study carried out with Chinese children (Zhao et al., 2020). The higher-order model is presumed to consist of one overarching factor of relational reasoning that is not represented by but significantly linked to the four factors representing each of the scales. These four scales are comprised of the eight items loading onto each scale. The proposed three models were tested using confirmatory factor analysis techniques with model-data fit indices. The confirmatory factor analysis was performed on the entire sample.

**FIGURE 3:**
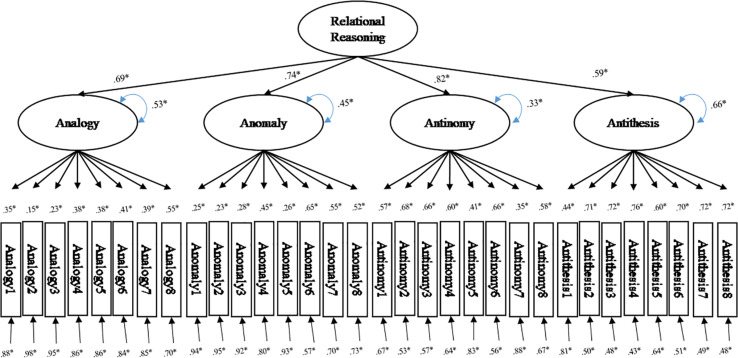
Higher-order factor model with standardized coefficients; *p < 0.05.

The confirmatory factor analysis showed that the one-factor model did not fit the data well. However, the four-factor model and the higher-order model were fit for the data in this investigation (Table 5). Specifically, the four-factor model and higher-order model produced the smallest values for Root Mean Square Error of Approximation (0.031), highest values for Comparative Fit Index (CFI; 0.933, 0.932) and Tucker-Lewis Index (0.927, 0.926). This trend was consistent for data with grades 4 and 6 and the models appeared to marginally fit data well according to rules of thumb (Schermelleh-Engel et al., 2003). Data with grades 8 and 10 also favored the four-factor model and higher-order model, but did not reach the goodness of fit standards. As a result, we concluded that the higher-order factor model was the best fit for the TORRjr data for these Korean students due to the fitness indices and the previous theoretical configurations (Figure 2C). In addition, the higher-order model was more parsimonious (*df* = 460) than the competing four factor model (*df* = 458). This outcome parallels with findings from the calibration study (Zhao et al., 2020). Figure 3 shows the coefficients that were observed from the relational reasoning structure with the Korean samples in grades 4, 6, 8, and 10. It was indicated that all 32 items loaded onto the appropriate scale of the TORRjr. For example, the eight analogy items loaded onto the analogy scale, while the antinomy items loaded onto the antinomy scale. Further, the four forms significantly loaded on the highest-order, relational reasoning factor.

**TABLE 5:** Fit indices for the one-factor, four-factor and higher-order model- by grade cluster.

Grade Cluster	Fit Indices				
**Model**	**χ *^2^***	***df***	***CFI***	***TLI***	***RMSEA***
Grades 4–10					
One-factor	2045.06	464	0.68	0.66	0.07
Four-factor	793.68	458	0.93	0.93	0.03
Higher-order	800.60	460	0.93	0.93	0.03
Grades 4–6					
One-factor	1328.84	464	0.62	0.59	0.07
Four-factor	617.07	458	0.93	0.92	0.03
Higher-order	620.02	460	0.93	0.92	0.03
Grades 8–10					
One-factor	1310.27	464	0.67	0.65	0.07
Four-factor	758.27	458	0.88	0.87	0.04
Higher-order	761.72	460	0.88	0.87	0.04

**CFI*, comparative fit index; *TLI*, Tucker-Lewis Index; *RMSEA*, root mean square error of approximation.*

### Research Question 2: Measurement Invariance Test

The contribution of grade level and gender to students’ relational reasoning performance measured by the TORRjr was investigated by measurement invariance test (Tables 6, 7). Specifically, using the measurement invariance function in R (Rosseel and Jorgensen, 2019), we tested four models that progressed from least to most constrained. In the least constrained model (*configural invariance*, Model 1), the paths for all psychometric components (i.e., factors loadings intercepts, and latent means) are presumed to vary by gender or by grade level. In contrast, for the most constrained model (*strict invariance*, Model 4), it is presumed that all psychometric components would be the same regardless of gender or grade. For Model 2 (*metric invariance*), the factor loadings are assumed to be equivalent for the boys and girls and for fourth, sixth, eighth, and tenth graders. In contrast, in Model 3 (*scalar invariance*), factor loadings and intercepts are expected to be similar for males and females and grade level.

**TABLE 6:** Measurement invariance test of gender: model comparisons in higher-order structure for grades 4–10.

Model	Constraints	*df*	*AIC*	*BIC*	χ*2(Δχ2)*	Δ*df*	*Pr(>χ ^2^)*		*CFI*	Δ*CFI*	Compared to
1. Configural Invariance	No	920	22375	23289	1298.1				0.920		
2. Metric Invariance	Factor Loadings	952	22333	23100	(21.478)	32	0.921		0.923	−0.003	Model1
3. Scalar Invariance	Factor Loadings and Intercepts	979	22317	22962	(38.559)	27	0.070	.	0.920	0	Model2
4. Strict Invariance	Factor Loadings, Intercepts, and Latent Means	984	22324	22945	(16.740)	5	0.005	**	0.918	0.002	Model3

*AIC*, Akaike’s Information Criteria; *BIC*, Bayesian Information Criteria; Smaller *AIC* and *BIC* are desirable; *CFI*, Comparative Fit Index; Larger *CFI* is desirable. *p < 0.05; **p < 0.001; ***p < 0.0001.

**TABLE 7:** Measurement invariance test of grade level: model comparisons in higher-order structure for grades 4–10.

Model	Constraints	*df*	*AIC*	*BIC*	χ*2(Δχ2)*	Δ*df*	*Pr(>χ ^2^)*		*CFI*	Δ*CFI*	Comparison to
1. Configural Invariance	No	1840	22942	24789	2670.9				0.841		
2. Metric Invariance	Factor Loadings	1936	22907	24311	157.22	96	8.189E-05	***	0.829	0.012	Model1
3. Scalar Invariance	Factor Loadings and Intercepts	2017	22907	23937	162.34	81	2.189E-07	***	0.814	0.027	Model2
4. Strict Invariance	Factor Loadings, Intercepts, and Latent Means	2032	23002	23962	124.54	15	<0.0001	***	0.793	0.048	Model3

*AIC*, Akaike’s Information Criteria; *BIC*, Bayesian Information Criteria; Smaller *AIC* and *BIC* are desirable; *CFI*, Comparative Fit Index; Larger *CFI* is desirable. *p < 0.05; **p < 0.001; ***p < 0.0001.

#### Gender Difference

For the test of measurement invariance for gender, we first eliminated data for 35 missing values on gender and then ran analyses for four models previously described. As the fit statistics in Table 6 indicate, Model 3, representing *scalar invariance*, was the most statistically viable model. Specifically, a significantly worse change appeared at *scalar invariance* (Model 3) but not at *configural invariance* model (Models 1) and *metric invariance* model (Model 2) according to the chi-square changes by step ($\chi_{d\,i\,f\,f}^{\text{2}}$ = 16.74, *p* = 0.005) and the proposed ΔCFI cutpoint of 0.01 (Cheung and Rensvold, 2002). The significant change from *metric invariance model* to *scalar invariance model* means that there was measurement invariance for the factor loadings and intercepts for the male and female students in this investigation. This *scalar invariance* model is considered a satisfactory condition for measurement invariance (Bialosiewicz et al., 2013; Milfont and Fischer, 2015). Therefore, for the Korean students in this study, the TORRjr was determined to be invariant with regard to gender.

#### Grade Level Difference

For the measurement invariance test for grade level, we determined that none of the four models fit the data well. However, the *configural model*, in which all paths are presumed to vary, was shown to fit the data best of the four models. Specifically, as displayed in Table 7, our chi-squared model-fit indices already began to show significant χ*^2^* changes from Model 1 (*configural model*) to *metric model* (Δχ*^2^* = 157.22, *p* < 0.0001). In other words, the *metric model* was significantly worsened by adding more constraints to the *configural model*. Thus, although the higher-order factor structure functioned well for all grade levels, there were differences in the performance of the students in grades 4, 6, 8, and 10 at the scale and item levels. Such differences might be expected given the age ranges included in the study and in light of the pattern in relational reasoning development described previously. This outcome supports conducting separate standardizations of the TORRjr for the younger (grades 4 and 6) and older (grades 8 and 10) students.

### Research Question 3: Suitability of TORRjr for Grades 8 and 10

**FIGURE 4:**
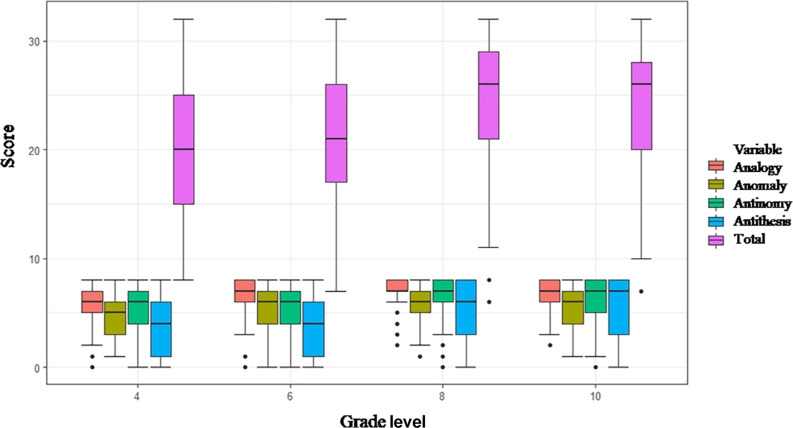
Boxplot of the TORRjr scores by relational reasoning form and grade level.

Whether the TORRjr is a psychometrically appropriate measure of relational reasoning for older students (grades 8–10) compared to younger students (grades 4–6) was tested above section in line with gender effect using measurement invariance test. Accordingly, a significant difference was evident between younger students’ and older students’ relational reasoning. Specifically, older students performed significantly better at the TORRjr than younger students did. This study finding parallels with the findings from Zhao et al. (2020). The use of the TORRjr was originally suggested up to grade 7. As we noticed in Figure 4, the distribution of relational reasoning scores was close to normal in grades 4, 6, and 8. However, a ceiling effect was apparent in the composite score of the TORRjr in grade 10 (see Figure 4). Given this trend, grade 8 seems to be the boundary for using the TORRjr. For grade 10, using the TORR seems preferable to using the TORRjr.

### Research Question 4: Trends by Grade Levels

To explore the trends of overall TORRjr performance and the four individual scales in grades 4, 6, 8, and 10, we conducted an additional scale-level analysis using non-parametric median-based tests due to the non-normality of the data, as presented in Table 8. Specifically, we ran the Kruskal–Wallis test to compare the four age groups and the Mann-Whitney *U* test for *post-hoc* analysis.

**TABLE 8:** Mann-Whitney *U* (MWU) test – pairwise comparisons.

Form (item numbers)	Grade 4 vs. Grade 6				Grade 6 vs. Grade 8				Grade 8 vs. Grade 10			
					
	*MWU*	*Z*	*p*		*MWU*	*Z*	*p*		*MWU*	*Z*	*p*	
Analogy (1∼8)	14800	−4.412	0	***	−2.403	−2.402	0.016	*	2.326	−2.260	0.024	*
Anomaly (9∼16)	14915	−4.236	0	***	−2.364	−1.936	0.053		2.710	−2.640	0.008	**
Antinomy (17∼24)	19337	−0.322	0.747		−4.132	−4.162	0	***	0.616	−0.326	0.745	
Antithesis (25∼32)	19604	−0.084	0.933		−5.259	−5.209	0	***	−1.075	−1.295	0.195	
Total	17039	−2.330	0.020	*	−5.307	−5.230	0	***	1.039	−0.817	0.414	

*p < 0.05; **p < 0.001; ***p < 0.0001.

The use of a boxplot has been recommended to avoid missing important hidden information such as dispersion, the symmetry of data values, and outliers (Williamson et al., 1989). It should be noted that 5 non-responding students were excluded from the actual analysis because we assumed eight or more consecutive non-responses meant these failed to complete one entire scale on the TORRjr. The boxplot (Figure 4) shows that the median scores increased to grade 8 and remained at a similar level from grades 8 to 10. The tenth graders’ scores were more negatively skewed than those of their younger counterparts. This suggests that the ceiling effect is more likely to apply to these older students.

When scale-specific analyses were conducted, the data skewness was found to be more apparent for the more complex scales. For example, the medians of the analogy scale were 6 and 7 and those of the anomaly scale were 5 and 6 at grades 4 and 6. The median of both the analogy and anomaly scales was 7 at grades 8 and 10. In contrast, the differences between the grade levels were more considerable for the antinomy and antithesis scales. The developmental trajectory was most apparent for the antithesis scale. Specifically, the medians for the antinomy scale were 6, 6, 7, and 7 for the students at grades 4, 6, 8, and 10, respectively, while the medians for antithesis scale were 4, 4, 6, and 7 for those same grades. The modes for antithesis were 1, 1, 8, and 8, respectively. In other words, the antithesis scale scores were lower at the lower age levels and increased up to grade 10, again suggesting a developmental trajectory.

The non-normality of the data required the use of nonparametric tests that rely on the median (i.e., Kruskal–Wallis test) rather than the mean. This analysis revealed significant group differences in the median scores of all the four forms of relational reasoning: analogy (χ^2^ = 47.604, *df* = 3, *p* < 0.001), anomaly (χ^2^ = 43.597, *df* = 3, *p* < 0.001), antinomy (χ^2^ = 31.414, *df* = 3, *p* < 0.001), antithesis (χ^2^ = 63.080, *df* = 3, *p* < 0.001), and composite (χ^2^ = 74.022, *df* = 3, *p* < 0.001). In other words, the differences across the age groups were most prominent for antithesis. The Mann-Whitney *U* test was conducted as a *post-hoc* test (Table 8). Significant differences emerged between grades 4 and 6 on analogy, anomaly, and the total score, between grades 6 and 8 on analogy, antinomy, antithesis, and the total score, and between grades 8 and 10 on analogy and anomaly.

In sum, there was a significant increase in analogy and anomaly from grades 4 to 6. There was a marginally significant development in analogy and a significant development in antinomy and antithesis from grades 6 to 8. Further, there was a marginally significant development in analogy and anomaly from grades 8 to 10. These changes across the grade levels indicate that development of relational reasoning ability, as measured by the TORRjr, does occur.

## Conclusion and Implications

In this study, we examined the development of relational reasoning for South Korean students in grades 4, 6, 8, and 10 using the TORRjr. The results of this study have garnered new insights about the nature of relational reasoning and the viability of the TORRjr as a measure of this fundamental cognitive ability for a yet untested population. However, before we summarize those discernments, there are certain limitations that we must acknowledge.

### Limitations

One major limitation of the current investigation concerns the representativeness of the student sample. Specifically, the students who participated were recruited from schools in Gangwon-do, one of South Korea’s nine provinces. Therefore, it cannot be concluded that these 749 students’ performance is representative of students’ performance nationally. Moreover, this study included only the TORRjr and did not incorporate any additional cognitive or academic measures to serve as indicators of convergent, discriminant, or predictive validity. It will be essential to design subsequent studies that allow for more comprehensive validation of the TORRjr with a nationally representative sample of students in grades 3 to 7 – the range of the grade levels for which the TORRjr was initially designed and at which the test seemingly performs optimally for normally developing students.

### Key Findings

Despite the aforementioned limitations, important insights into children’s and young adolescents’ relational reasoning were garnered from this investigation.

#### Psychometric Properties

First and foremost, the present study demonstrated the viability of the TORRjr as a measure of relational reasoning for South Korean students, particularly those in grades 4 and 6. Even though the data for the students in grades 8 and 10 were acceptable at the item and factor levels, the distribution of data for these upper grades was non-normal with high skewness and kurtosis. This suggests that these older students might be better served by the TORR, which was developed expressly for adolescents and adults.

Across all four grades, the item difficulties were determined to be within the acceptable range. However, certain items seem somewhat easier for the students in this study than in the prior research with Chinese children in grades 3–7 (Zhao et al., 2020). Further, the higher-order factor structure held for performance at both the lower grade levels and the upper-grade levels in both the current and prior investigations. Also, the higher-order factor model that emerged in this investigation as the best fit of the data mirrored the model found previously for the TORRjr (Zhao et al., 2020) as well as for the TORR (Dumas and Alexander, 2016).

With regard to measurement invariance, the current study revealed grade level is a source of measurement *variance* but gender is not. The variance due to grade level seems to partially related to the participants’ developmental patterns as we discuss later in this section. In other words, the participants might tend to be measured differently by their grade levels because they were developed the way their age group was supposed to be developed. In contrast, the measurement invariance seems little to do with their gender. This invariance by gender opens up more discussions due to its inconsistency with previous investigation (Zhao et al., 2020) although the measurement invariance captured in this study is more promising for the use of TORRjr in the field. Only according to the current study result, we can conclude TORRjr is a fair measure for relational reasoning in any gender.

#### Developmental Trajectories

Once the viability of the TORRjr was established for the current sample, a primary purpose of this study was to explore the developmental path of relational reasoning ability from students in grades 4 to 10. We wanted to explore the trajectory based on the overall performance on the TORRjr and performance on the individual scales. As noted in the theoretical framing, the developmental path for relational reasoning ability portrayed in the literature is unresolved. Some scholars have documented a decline in relational reasoning ability during adolescence (Carey et al., 1980; Diamond et al., 1983; Dumontheil et al., 2010), whereas others contend that the developmental path varies for the different forms of reasoning considered (Jablansky et al., 2016, 2019; Zhao et al., 2020).

In this study, the developmental path for the TORRjr total score across grades 4, 6, 8, and 10 followed an asymptotic course, with a level off witnessed from grades 8 to 10. Such a finding seems to give credence to studies that reported a decline in relational reasoning ability during adolescence (Dumontheil et al., 2010). However, the developmental trajectory followed a different path when the lower grades (fourth and sixth) and upper grades (eighth and tenth) were tracked separately and when scale scores rather than a total score were the focus of analysis. When those changes were made, what emerged was a gradual and significant improvement from grades 4 to 6 and from 6 to 8. Moreover, the leveling off we observed between grades 8 and 10 seemed primarily attributable to the ceiling effect for the TORRjr within the older participants, rather than a decline in relational reasoning ability *per se*.

Even more enlightening were the shifts that occurred in the TORRjr scales over time. In this study, as with others investigating the four forms of relational reasoning (Jablansky et al., 2019; Zhao et al., 2020), the analogy scale was the easiest for students at every grade level, followed by the antinomy scale. This is particularly important because the studies of relational reasoning that argue for a decline of this foundational ability in adolescence have only tested analogical reasoning.

The picture is far more complicated when antinomous and antithetical reasoning are added to the mix. Specifically, while younger students in this study rely more on analogical and antinomous reasoning, as in prior investigations (Jablansky et al., 2016, 2019; Zhao et al., 2020), older students manifest increasing reliance on antinomous and antithetical reasoning. Thus, there is the continued development of relational reasoning ability as measured by the TORRjr, even among older students, but evidenced more by certain forms of reasoning. Why do antinomous and antithetical reasoning follow a somewhat different developmental path than analogical and anomalous reasoning? For one, these forms of relational reasoning appear to be less familiar and more cognitively demanding. As seen in the sample problems in Figures 1C,D, solution of these items entail multiple steps to solve the problems. Specifically, with the antinomous scale, respondents must determine what attributes define the given problem set. Then they must test the various options presented to ascertain which of the options have no elements in common with the given problem set.

Similarly, respondents to the antithesis items must first grasp the conversion process depicted in the given problem and then reverse that process to find the correct option. Overall, the performance of the four scales by younger and older students in this study suggests that relational reasoning ability continues to develop into adolescence. Yet, some of the various reasoning forms have an earlier onset than others. Therefore, the continued reliance solely on analogical reasoning as a relational reasoning marker effectively masks significant developmental patterns in this foundational cognitive ability.

### Future Directions

While the current study unearthed several significant findings about relational reasoning and its development for South Korean elementary and middle-school students, there is unquestionably more work to be done. We identified several future studies that represent critical next steps in this research venue within the summary of key findings from this investigation. Those future studies include the standardization of the TORRjr using a representative national sample of South Korean students in grades 3 to 7 and incorporating cognitive and academic measures that permit the assessment of the TORRjr’s convergent, discriminant, and predictive validity.

Additionally, it would be worth considering the effects of relational reasoning on the academic performance and development of specific atypical populations among the student population. For instance, it would be invaluable to understand the role that relational reasoning plays in the learning of various “identified” populations such as students with learning disabilities or those dealing with subject-specific problems in reading or mathematics. Within South Korean schools, there are also students who are significantly older than their grade-level peers, due to events that disrupted or inhibited their formal education. Examining the performance and development of relational reasoning abilities of these atypical student populations may provide critical insights into their learning patterns and afford suggestions for instructional programs to assist in their academic development.

Finally, the insights garnered from this study were based on cross-sectional data. If the development in relational reasoning is to be more fully understood, it is imperative that a longitudinal investigation be undertaken. Further, in light of the shifts in reasoning that occurred in students we tested around grade 8, it seems advisable to follow groups of students in grades 4 and 6 over 2 years. To our knowledge, this would be the first such longitudinal study of the TORRjr, and it would afford rich profiles of reasoning during critical periods of neurocognitive development.

Although there is so much to learn about the nature and development of relational reasoning abilities, we regard the present study as vital steps forward. Given what has already been demonstrated about the foundational role that relational reasoning plays in learning and academic performance within a wide array of fields – from engineering and mathematical thinking to medicine and literacy (e.g., Dumas et al., 2014; Dumas and Schmidt, 2015) – it is undoubtedly a worthy empirical pursuit.

## Data Availability Statement

The raw data supporting the conclusion of this article will be made available by the authors, without undue reservation.

## Ethics Statement

The studies involving human participants were reviewed and approved by Gangneung-Wonju National University. Written informed consent to participate in this study was provided by the participants’ legal guardian/next of kin.

## Author Contributions

Both authors listed have made a substantial, direct, and intellectual contribution to the work and approved it for publication.

## Conflict of Interest

The authors declare that the research was conducted in the absence of any commercial or financial relationships that could be construed as a potential conflict of interest.
